# She Thought It Was Her Change of Life

**Published:** 1890-07

**Authors:** A. Vander Veer

**Affiliations:** Albany, N. Y.; Professor of Clinical, Didactic, and Abdominal Surgery in Albany Medical College; Fellow of the American Association of Obstetricians and Gynecologists, etc.; 28 Eagle Street


### Buffalo Medical and Surgical Journal

Vol. XXIX. JULY, 1890. No. 12.

### Original Communications.

### "SHE THOUGHT IT WAS HER CHANGE OF LIFE."

REPORTING CASES OF UTERINE POLYPI, UTERINE CANCER, INVERSION OF THE UTERUS, AND SUB-PERITONEAL FIBROID.

By A. VANDER VEER, M. D., Albany, N. Y.

Professor of Clinical, Didactic, and Abdominal Surgery in Albany Medical College; Fellow of the American Association of Obstetricians and Gynecologists, etc.

The object of this paper is to concentrate upon certain cases that occur in our consultation practice, and in the practice of the overworked general practitioner, a closer and more positive examination for the purpose of a correct diagnosis. I will proceed at once to the report of a case that will help to illustrate what I have in mind:

Miss M., aged 47, a well-to-do maiden lady, residing in a small country town, had been in good health, regular in her menstruation up to the age of 44, when she began to flow more excessively at her menstrual periods, and soon thereafter developed a condition of both menorrhagia and metrorrhagia After this condition had lasted for a year, and when she was quite anemic, somewhat exhausted, and losing in flesh, she consulted her family physician, Dr. B., who treated her for some time with tonics, rest, and diet, with some little benefit, but not much improvement as to the excessive flowing. He suggested making a careful examination as to her condition, but this she positively refused to have done, saying that "she believed it to be only her change of life." He treated her for another year, at the end of which time she was confined to her bed, and yet refused to have any local treatment. When she had suffered for nearly three years, and in a condition in which there was much edema of the extremities, her lips colorless, and a profound state of anemia present, at the earnest solicitation of the members of her family she finally yielded, and the doctor was permitted to make an examination which confirmed his previous suspicion of a uterine fibroid. It presented in the form of a simple polypus projecting from the external os. Her condition was made known to her, and an immediate operation urged. I was sent for on October 10, 1887, and found her in such an exceedingly feeble condition that I really feared she might die from the additional slight shock of the operation. She, however, was very willing to have done what seemed to be best, was now entirely passive, knowing that she could live only a short time if not helped in some way. I found a large polypus filling the cavity of the vagina, and attached by a moderate-sized pedicle to the internal os. Around it I was able to pass the chain of the écraseur, and to remove it without any great trouble, not giving the patient an anesthetic, as I feared she would be unable to endure it. The cavity of the uterus was curetted thoroughly, washed out with a weak solution of bichloride, and packed with iodoform-gauze. This was removed at the end of forty-eight hours, and afterward vaginal douches made use of, containing boracic acid in solution. This patient ultimately made a complete recovery, although her convalescence was somewhat slow in consequence of her exceedingly weak and exhausted condition.

I present this as a case familiar to many of us, illustrating a class of cases where procrastination on the part of the practitioner, with absolute indifference and stubborness on the part of the patient, often costs the latter her life. Women seem to have in their mind the idea that they must expect all sorts of conditions to present at the time of the menopause, and are over-negligent, too frequently, in having their cases properly looked into.

Belonging to another class of cases, which are far more sad, is the following:

Mrs. B., aged 33, married, mother of three children, has always been in good health, family history good, youngest child three years of age, which she nursed and weaned at the age of fourteen months; menstruated regularly after that until six months previous to her admission into the Albany Hospital, September, 1886. During that time, her flowing had continued almost constantly; she suffered little pain, but was much weakened; had emaciated somewhat, yet continued attending to her household duties, refusing all local treatment. Her family physician finally told her that he would have nothing more to do with her case, and that she must go to the hospital, where she came under my care. On making an examination, I found an epithelioma that embraced the entire cervix, extending to the lateral walls of the vagina, to the under surface of the neck of the bladder, and extending up along the lower portion of the urethra. It was absolutely impossible to do anything for her in the way of treatment or operation, and when she was informed of her true condition the sadness of the scene is but too well known to many of us.

This illustrates a class of cases by far too numerous, as they present in hospital practice, and yet, notwithstanding the time of life at which this patient complained, she, too, insisted that "she supposed it was her change of life, and that she would soon be all right."

Belonging to still another class of cases are some such remarkable ones as I here report, where, having passed the menopause in a normal manner, the patient afterward presents a condition of flowing and exhaustion, but still entertains the idea that it is simply a return of the menstrual flow, and which indicates another phase or condition of change. The following cases illustrate this somewhat:

Mrs. G., aged 65, married, native of Canada, mother of seven children, a strong and healthy woman all her life, passed her menopause, without any unusual symptoms, at the age of 49. At the end of three years, during which time she had been in good health, she began, as she supposed, her menstruation again. She did not pay very much attention to it at first; it came on at irregular intervals and continued so, at times flowing very severely. During March and April, 1889, she visited Chicago, when, flowing very severely, and being under the care of a physician of the family where she was stopping, after examination, she was told by him that she had a uterine polypus, and that he would operate upon her by dilating the womb and removing it. She did not like to be operated upon away from home, was fearful of the effect of an anesthetic, and returned to her family. Her flowing continued at intervals, with more than usual severity, accompanied with very much pain at times. She described her pain as being of an expulsive character, not unlike that of child-bearing, as she stated. The pain during July and August was unusually severe, and she realized that something was projecting from the vaginal orifice. About August 15, 1889, this became very prominent and somewhat offensive. She had been treated by her local physician, who failed to make any diagnosis of the case. On August 23d, Dr. Turner, of Crown Point, N. Y., was called to see her, and was somewhat startled, on entering the room, to notice the very marked odor of gangrene that presented. On examination, he found a mass protruding from the vulva, the exact character of which he was unable to diagnosticate. I was telegraphed for, but did not see her until August 26, 1889. I then found the mass protruding, as seen in the accompanying specimen. After a thorough and careful examination, I reached the conclusion that she had been suffering from uterine polypus, which had gradually extruded itself from the cavity, bringing down the fundus of the uterus, and causing inversion of the same. I could feel the lips of the external os well up in the vagina. Taking all things into consideration,—her age, and the nasty gangrenous condition of the presenting mass,—I concluded that it was not wise to dissect off the polypus, and re-invert or return the uterus, but to throw around that portion of the fundus that could be easily reached the chain of the ècraseur, and remove the mass in that manner. She bore the operation without taking an anesthetic; the hemorrhage was not at all copious; the parts were thoroughly douched with a bichloride solution, and the cavity of the vagina packed with strips of iodoform-gauze. These were removed at the end of the second day, and afterward the vaginal douche of boracic acid solution was continued daily. The specimen, as you will observe, contains the right horn of the uterus and Fallopian tube, the ècraseur having reached well above the sloughing mass. She made a good recovery, and is now in excellent health.

The next is a case quite as remarkable in many respects:

Mrs. B., aged 72, married, mother of three children, her husband a physician, but who had been in a very sad condition of nervous prostration for a period of ten or fifteen years. Mrs. B. had always enjoyed good health, but at the time of her menopause flowed very severely and irregularly. She supposed that she had ceased to flow at the age of 53, and was in fair health for a few years; but, feeling some distress later on, consulted the late Dr. Goldsmith, of Rutland, Vt., who told her that "she had falling of the womb," and fitted her with a glass pessary, which she wore without removing for fifteen years. She could then retain it no longer, and suffered much for the following year. Finally, she consulted another physician, and an attempt was made to have her wear, first a Babcock external supporter with stem pessary, and, later, a MacIntosh, all of which were utterly useless. She suffered a right hemiplegia two years ago, when seventy, from which she made a good recovery. Six months ago she noticed, as she supposed, an entire prolapse of the uterus, which she could press back with much difficulty, and which she continued to do until about two months ago, when she failed to return it. It now remained out, she was confined to her bed, gradually growing worse, but her husband not in a condition of mind to recognize the serious state of her health. Her son, a very competent physician, she did not consult, although he saw her daily, until about January 28, 1890, when, noticing her condition, the odor of the room, etc., he made careful inquiry of his sister, and then, for the first time, learned the serious condition of his mother. He immediately sent for one of his neighboring physicians, who made an examination, but was unable to state positively what he believed to be the real trouble. February 1, 1890, the doctor called at my office desiring me to see his mother at once. I did so the following day and found a sloughing fibroid protruding from the vulva, presenting the most offensive odor possible. The room had been kept thoroughly ventilated, but the odor was almost unbearable, and the patient seemed much distressed and in a very anxious condition of mind. She stated that she supposed for a long time that her flow had returned, and that she did not think there could be anything seriously wrong until the mass protruded from the vulva. The fibroid had its attachment to the anterior wall of the uterus, and, very curiously rested, between the cervix and posterior wall of the bladder. By passing the catheter into the latter viscus I obtained a very correct idea of the surroundings and concluded to remove it with the chain écraseur, which I did with little trouble. After removal, the uterus returned to its position. She made an uninterrupted recovery, and is again able to care for her invalid husband.

The point that I wish to present is this: that these cases are to be found all over the country, and that in some way and in some manner we should indicate to our patients the importance of their yielding to a more prompt examination, when such histories present as are here given. Our young women should be taught in our schools, academies and colleges, more on the subject of menstruation. They should learn more about their reproductive organs from chaste, moral, and intelligent teachers. Mothers should know more of the functions of their own individual organs, and learn to teach their daughters.

Finally, the profession should exercise more care in impressing upon young wives and mothers the knowledge that in so many cases they so sadly need, and not assume the care of patients who are so unwilling to have the necessary examination made.

28 Eagle Street.

---

Philadelphia is said to contain about one million people more comfortably cared for than the people of any other city. Yet it is also said that two hundred and fourteen thousand of these receive pauper medical services. This is exclusive of private dispensaries, or the multitude treated by doctors free in their private practice. All this is due to the fact that doctors have taught the people that they desire to work for nothing.—*American Lancet.*

